# Wetting-Based Comparison of Ag, Carbon Black, and MoS_2_ Composite Membranes for Photothermal Membrane Distillation

**DOI:** 10.3390/membranes13090780

**Published:** 2023-09-04

**Authors:** Tarik Eljaddi, Corinne Cabassud

**Affiliations:** Toulouse Biotechnology Institute, Université de Toulouse, CNRS, INRAE, INSA, 135 Avenue de Rangueil, CEDEX 04, 31077 Toulouse, France

**Keywords:** photothermal membrane distillation, wetting, desalination, composite membrane, carbon black, MoS_2_, Ag, plasmonic particles

## Abstract

Photothermal membrane distillation is a new-generation desalination process that can take advantage of the ability of specific materials to convert solar energy to heat at the membrane surface and thus to overcome temperature polarization. The development of appropriate photothermal membranes is challenging because many criteria need to be considered, including light to heat conversion, permeability and low wetting, and fouling, as well as cost. Based on our experience with wetting characterization, this study compares photothermal membranes prepared using different well-known or promising materials, i.e., silver nanoparticles (Ag NPs), carbon black, and molybdenum disulfide (MoS_2_), in terms of their structural properties, permeability, wettability, and wetting. Accordingly, membranes with different proportions of photothermal NPs are prepared and fully characterized in this study. Wetting is investigated using the detection of dissolved tracer intrusion (DDTI) method following membrane distillation operations with saline solutions. The advantages of MoS_2_ and carbon black-based photothermal membranes in comparison with polyvinylidene difluoride (PVDF) membranes include both a permeability increase and a less severe wetting mechanism, with lower wetting indicators in the short term. These materials are also much cheaper than Ag NPs, having higher permeabilities and presenting less severe wetting mechanisms.

## 1. Introduction

Photothermal membrane distillation (PMD) is a promising solar-driven process for producing drinking water in remote locations. The first application of photothermal membranes for seawater desalination was recently proposed [1,2], and several recent reviews have focused on this topic [3,4,5,6]. PMD is a membrane distillation (MD) process that uses specific membranes incorporating photothermal nanoparticles. By definition, photothermal materials can convert solar energy to thermal energy thus contributing to the heat requirement for MD operation. Photothermal materials are based on the following principle: according to quantum theory, when photons collide with electrons, some energy can be absorbed by the electron and this absorbed energy can be released via various processes including as thermal energy [1].

In PMD, the feed is separated from the distillate by a hydrophobic microporous membrane, as in conventional MD. Under sunlight irradiation, the photothermal active layer provides localized heating at the evaporation surface [2,3]. The PMD operation involves three main mechanisms: (i) photothermal conversion of light to heat, an additional benefit in comparison with MD; (ii) vaporization, which transforms water into vapor that then diffuses inside the membrane pores, such that a liquid–vapor interface should be maintained at pore inlets to avoid pore wetting and to guaranty a good quality permeate; and (iii) condensation, which transforms water vapor into the liquid phase [4]. 

One of the main advantages of using PMD is that, in comparison with MD, temperature polarization can be overcome because the presence of photothermal nanoparticles (NPs) in the polymeric membrane allows a reversal of the temperature profile close to the membrane and a higher temperature to be maintained at its surface. This further enables a higher evaporation rate, mass transfer through the membrane pores, and a higher permeate flux [3,5]. Because temperature polarization is a limiting phenomenon in MD, PMD is an interesting alternative to better use heat energy and to enhance the water productivity. However, several other phenomena can contribute to limiting the PMD efficiency, including membrane wetting, fouling, and ageing, and the development of PMD requires additional research concerning the choice of photothermal material and the manner in which these materials are included inside the membrane matrix. Moreover, when desalination is the envisioned process, the cost of the membranes and therefore of the photothermal materials is an important consideration.

Four families of photothermal materials, corresponding to different mechanisms of converting solar energy to heat energy, have been widely investigated for photothermal membrane development for different types of membrane processes (Table 1). The physical mechanisms ruling light to heat conversion in PMD are not yet fully understood, but have been explored for some photothermal materials, such as metallic nanostructures [7]. More details on mechanisms can be found in the literature [4,5,6,7,8,9].

The application of photothermal materials to PMD for desalination is a very recent topic of research. So far, the most-often investigated NP materials in this area have been Ag [1,7,8], Au [10,11,12], TiO_2_ [13], Fe_3_O_4_ [14], carbon black [15], carbon nanotubes [16], and graphene [17]. In previous studies, very often only the evaporation rate under light has been measured but not the permeate flux achieved in PMD operation. To the best of our knowledge, the highest fluxes in PMD have been reported for Ag NPs in polyvinylidene difluoride (PVDF) membranes [1]. Ag NPs generate a heat flux approximately 10 times higher than that achieved with gold NPs under plasmon resonance conditions and are less expensive than gold [1,18]. Dongare et al. were the first to demonstrate the use of carbon black NPs embedded in electrospun polyvinyl alcohol and deposited on a conventional PVDF MD membrane [15] to increase the membrane surface temperature on the feed side and reduce the energy requirement. They obtained a flux of 5.38 kg m^−2^ h^−1^ and a solar efficiency of 20%. Recently, experiments have been conducted on the filtration or spraying of MoO_3−x_ (0 < x < 1) on a polymeric membrane to build a photothermal layer at the surface of the membrane [19], with results showing high evaporation rates (95%), comparable to those achieved with graphene (92%). Recent publications demonstrate the better plasmonic activity of 2D nanomaterials such as transition metal dichalcogenides, notably MoS_2_ as a photothermal material. Chou et al. report that the single-layer MoS_2_ nanosheets not only show good water dispersibility, huge specific surface area, and good biocompatibility but also have good molar extinction and high light-to-heat conversion capability [20], and depositing a layer of this material on an MD membrane has shown promising results in terms of the evaporation rate [21].

However, further studies are required to develop photothermal membranes based on the consideration of different constraints for the sustainable operation of the PMD process. Such considerations include (i) the economic feasibility of the process, which is affected by the choice of particles, limiting the use of expensive materials such as noble metals or materials based on rare compounds; (ii) the risk of the release (long term or otherwise) of NPs into water during PMD, which should be avoided, considering the impact of these materials on human health; and, importantly, (iii) the integration of NPs into a membrane matrix or at its surface, which is likely to modify the membrane physicochemical and structural properties compared with a reference polymeric membrane without NPs.

Changing the membrane properties can affect the membrane wettability and wetting occurrence during PMD operation, which could render the PMD process non-sustainable. To avoid membrane wetting, the photothermal membrane must be hydrophobic with an appropriate porosity, pore size distribution, and membrane thickness. For example, Politano et al. found that the addition of Ag NPs can change the wettability indicators: the contact angle (CA) and the liquid entry pressure (LEP) [9]. In a previous study, based on a recently developed technique to study the wetting phenomenon in MD called the detection of dissolved tracer intrusion (DDTI) method [22], we showed the importance of considering wetting, defined by the intrusion of liquid water into membrane pores, in the early stages of the development of new PMD membranes to optimize the proportion of NPs in the membrane matrix [23]. This study focused on PVDF membranes containing Ag NPs (the most effective photothermal material in terms of permeate flux enhancement) and showed that, to avoid the wetting problem that can be caused by defects on the membrane structure, the proportion of Ag NPs should be lower than 25%.

In the present study, we compare the occurrence of wetting during MD for different membranes based on the same polymer (PVDF) and with the integration in the polymeric matrix of photothermal NPs made of different materials: Ag (highly performant in PMD but expensive); MoS_2_ (an emerging two-dimensional nanomaterial, promising in terms of the evaporation rate); and carbon black, the cheapest material that can be obtained by the valorization of organic waste in a circular economy. Accordingly, PMD flat-sheet experimental membranes were produced. The wetting mechanisms were then identified, and the wetting indicator (defined as the percentage of the pore length wetted) after MD operation with saline water was determined locally and globally using the DDTI method for each photothermal material as a function of its massic proportion in the membrane (from 0 wt%, corresponding to a pristine PVDF membrane, to 25 wt%).

## 2. Materials and Methods

### 2.1. Chemicals and Membrane Preparation

The chemicals used to prepare the composite membranes were a PVDF polymer (PVDF-761, Arkema, Lyon, France), MoS_2_ (Molybdenum(IV) sulfide, 90 nm, Sigma-Aldrich, Saint-Quentin-Fallavier, France), carbon black (100 nm, Sigma-Aldrich), a dimethylformamide (DMF) solvent (Sigma-Aldrich), and Ag NPs (Ag-100, 20 nm, Oocap SAS (https://www.getnanomaterials.com/, accessed on 12 July 2023), Saint-Cannat, France).

The membranes were prepared following the methods covered in Politano et al. [10] and Eljaddi and Cabassud [11]. Table 2 shows the quantities of the polymer (PVDF) and NPs and of the solvent (DMF) needed to prepare 100 g of collodion for the different target NP massic proportions (2%, 6%, 12%, and 25%). Flat-sheet membranes were prepared via phase inversion. The casting suspension was cast on a glass plate with an automatic film applicator machine (Elcometer, Manchester, UK) using a knife with a gap of 200 µm. The samples were then immersed immediately for 2 h at room temperature in a coagulation bath (tap water) and then in another coagulation bath (tap water) for 1 day to remove any trace of the solvent. The membranes were then dried in an oven at 60 °C for one night.

The following notation is used for the presented membranes: R denotes the PVDF-REF membrane; (M2, M6, M12, and M25) denote PVDF-MoS_2_ membranes; (C2, C6, C12, and C25) denote PVDF-carbon black membranes; and (A6 and A25) denote PVDF-Ag membranes, where the numbers (2, 6, 12, and 25) indicate the percentage of the given NP material in the membrane. For example, M6 is PVDF-MoS_2_ membrane with 6 wt% of MoS_2_.

The full results for the PVDF-Ag membranes have been presented and discussed in a previous paper [23]. Here, for comparison, we integrate the results achieved for 6% and 25% NPs, that is, the membranes denoted A6 and A25.

### 2.2. MD Pilot Plant

A schematic and the details of the vacuum membrane distillation (VMD) pilot plant used for the experiments can be found in our previously published studies [22,23]. The experimental MD cell allows the use of flat-sheet samples with an effective area of 4.16 × 10^−3^ m^2^.

The pilot plant was fed with pure water to determine the membrane Knudsen permeability (K_M_) and with a NaCl solution (35 g L^−1^) to determine the wetting indicator using the DDTI method. The corresponding operating conditions are given in Section 2.3. Ultra-pure deionized water (Milli-Q, conductivity < 3 µS) and sodium chloride (99.99%) purchased from VWR were used to prepare the pure water and saline solutions for the MD experiments.

### 2.3. Characterization of the Membrane Properties

#### 2.3.1. Structural Properties

SEM JSM-6400 (JEOL Europe, Croissy sur Seine, France) coupled with X-ray energy-dispersive spectrometry XFlash 6130 EDX (Bruker, Billerica, MA, USA) and the ES-PRIT 1.9 software was used to characterize the membrane morphology and to analyze the presence of NPs and Na and Cl elements on/in the membranes. For the cross-section analyses, samples were broken using liquid nitrogen and were observed via SEM/EDX after carbonization.

The membrane thicknesses were measured using an electronic micrometer (Schuts Model-134001), and the porosity was measured using a solvent method (ethanol) with a balance (Sartorius, Göttingen, Germany, 0.01 mg).

#### 2.3.2. Wettability Indicators

The contact angle and the liquid entry pressure are the main parameters used to characterize the membrane wettability, which characterizes the risk of wetting.

The contact angle characterizes the membrane surface hydrophobicity. The contact angle of the prepared membranes was measured using a drop shape analyzer (DSA25, Kruss, De (https://www.kruss-scientific.com/en, accessed on 12 July 2023)) and then analyzed using the ADVANCE software (https://www.kruss-scientific.com/en/products-services/advance-software/advance-drop-shape, accessed on 12 July 2023).

The liquid entry pressure of pure water (LEP_w_) is defined as the minimum hydrostatic pressure that must be applied to a membrane before water penetrates the largest pore of the membrane by overcoming the hydrophobic forces; this pressure can be detected on the permeate side [24,25]. The LEP_w_ measurements were performed using ultra-pure water at 20 °C. The experimental protocol to obtain LEP_w_ (Convergence) is based on the observation of the first drop of permeate during a pressure step filtration operation. This test was repeated three times, and the average value is reported for the membranes. More details concerning this test are provided in a previous publication [26].

### 2.4. Characterization of Membrane Performances

#### 2.4.1. Knudsen Permeability Coefficient

The Knudsen permeability coefficient, K_M_, can be used in VMD under our experimental conditions to characterize the membrane permeability to pure water during the MD process. K_M_ was obtained by applying the permeate pressure variation method with the VMD pilot plant. The following operating conditions were used: Feed = pure water; *T_feed_* = 42.5 °C, *P_permeate_* = 60 mbar; feed flowrate = 150 L h^−1^; and Re = 2200, where *T_feed_* is the temperature of the feed, *P_permeate_* is the pressure of the permeate, and Re indicates the Reynolds number. K_M_ was calculated using Equations (2) and (3) from Ref. [22]. To facilitate comparisons, K_M_ was also calculated at 20 °C.

#### 2.4.2. Evaporation Rate

**Figure 1:**
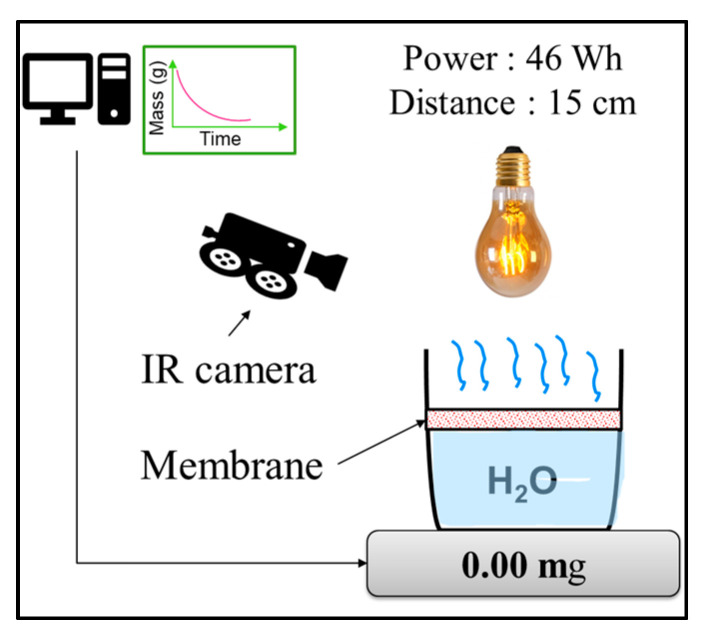
Evaporation test setup.

The plasmonic effects of the tested membranes were checked using a simple setup (Figure 1) allowing the evaporated water mass to be measured when the membrane was submitted to a fixed irradiation. A total of 50 g of distilled water recovered by each membrane sample was disposed in a Petri dish. The system was placed under a 46-Wh bulb held 15 cm from the membrane. The evaporated water mass was calculated over a 24-h period from the remaining mass, which was measured using a Sartorius balance (0.01 g) and recorded by a computer.

### 2.5. DDTI Method for Wetting Characterization

Wetting is defined by the intrusion of liquid water via convection through the membrane pores. Four theoretical wetting mechanisms are defined: no wetting; surface wetting; partial wetting; and total pore wetting [27] The DDTI method both identifies the wetting mechanism, from those theoretically defined, and provides a wetting indicator. The wetting mechanisms and indicators depend on both the membrane properties and the operating conditions [26,27].

In our previous study with Ag NPs [23], in addition to the DDTI method, we used an optical on-line method, which provides useful information concerning the wetting dynamics [24]. Unfortunately, this method cannot be applied to carbon black because of optical problems associated with the obtained black membrane.

Consequently, in this study, only the DDTI method was used to compare the membranes based on the different NPs (carbon black, MoS_2_ and silver). The DDTI method is based on the ex situ detection of the remaining traces of the intrusion of a tracer (salt) inside the membrane structure by SEM/EDX after the operation of the membranes in VMD with a saline solution (NaCl) under standard conditions. The DDTI method allows the wetting mechanism to be identified and the quantification of a wetting indicator (ω_p_), referred to as the pore wetting ratio, which represents the proportion of the membrane thickness into which liquid has intruded. ω_p_ can be calculated using Equation (5) from Ref. [22].

**Figure 2:**
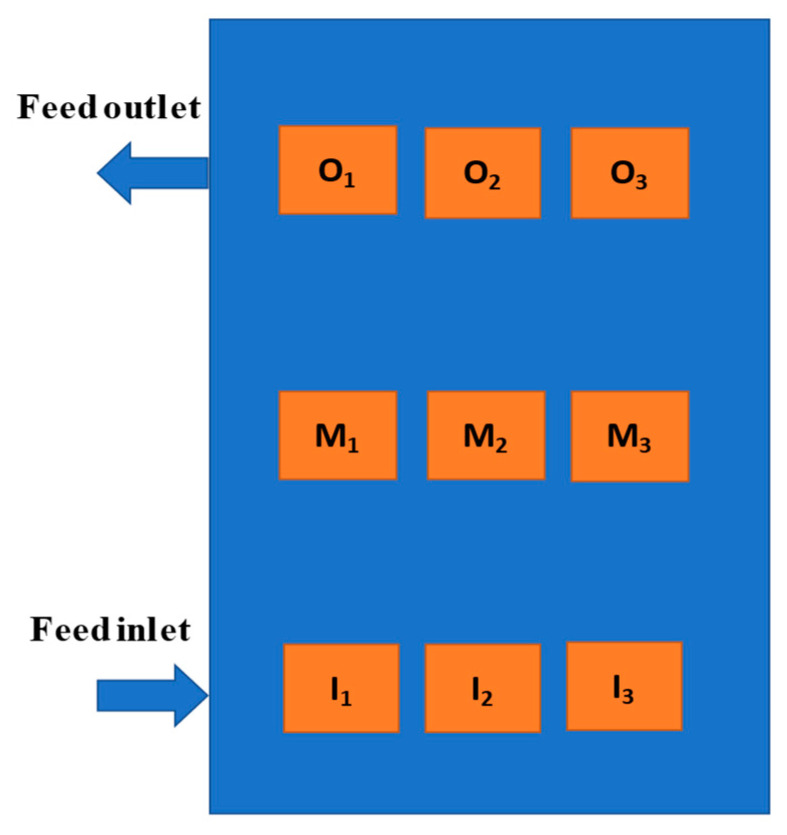
Sampling zones for the SEM/X-ray energy-dispersive spectrometry (EDX) analyses for wetting detection using the detection of dissolved tracer intrusion (DDTI) method.

The membranes were subjected to the VMD experiment with saline solutions with the same temperature, permeate pressure, and feed flowrate used in the permeability test (see Section 2.4.1) for 24 h. Then, the membranes were sampled and analyzed via SEM/EDX to detect traces of salt (NaCl) deposited on the surface or intruded into the membrane structure. The membranes were sampled without any cleaning (protocol B in Ref. [22]). The samples for the SEM/EDX analysis were taken in different zones of each membrane sample (Figure 2), as follows:

- Feed inlet: three samples (I_1_, I_2_, and I_3_);
- Middle of membrane: three samples (M_1_, M_2_, and M_3_); and
- Feed outlet: three samples (O_1_, O_2_, and O_3_).

The local wetting indicator ω_p_ of each sample was determined from the salt profile along the membrane thickness [22]. The average value of the local indicators in the nine sampling zones was used to determine the global ω_p_ value of each membrane. Given each salt tracer profile, ω_p_ was used to identify the wetting mechanism according to the correspondences shown in Table 3 with the code color to simply identification (Green color = no wetting, yellow color = surface wetting, orange color = partial wetting, red color = total wetting.

## 3. Results and Discussion

### 3.1. The Raw Materials

**Figure 3:**
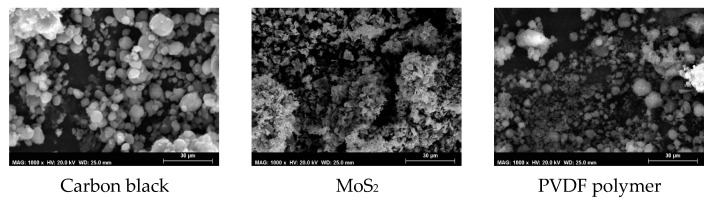
Scanning electron microscopy (SEM) characterization of carbon black, MoS_2_, and polyvinylidene difluoride (PVDF) at 1000× magnification.

Scanning electron microscopy (SEM) was used to characterize the raw materials, i.e., the carbon black, MoS_2_, and polymer particles, deposited on an observational support. Figure 3 shows that the polymer and carbon black particles have a spherical shape and that the MoS_2_ particles have a crystal-like shape. All raw samples show an agglomeration of particles.

### 3.2. Membrane Morphology and Structure

The SEM observations are shown in Table 4. The morphology of the pristine PVDF membrane corresponds to short finger-like structures with sponge substrates. It should be noted that membrane with Ag NPs A6 and A25 were prepared in previous work [23].

The same type of structure was obtained for the PVDF-Ag membranes A6 and A25 and by Yeow et al. with 15% PVDF (Kynar K760) in DMF [28]. The SEM observations clearly indicate that, for each type of NP material, the size of the finger structures increases with increasing NP proportion. The structure of the MoS_2_-based membranes presents a larger number of macrovoids when the NP load increases (from the M2 to M25 membranes). This was not observed for the carbon black-based membranes (C2–C25) for which sponge-like structures were more predominant than finger-like structures.

The results relative to the membrane thickness and porosity are given in Table 5.

- The membrane thickness is between 50 µm and 62 µm for both the NP materials and the loads. Overall, the thickness increased with the proportion of NPs from 2% to 25% for the MoS_2_ and carbon black membranes. Similar results have been reported for Ag NPs [23].
- The membrane porosity was between 57% and 62% for the reference and MoS_2_ and carbon black-based membranes, and the porosity can be considered as being within the same range for all three types of membrane. The Ag-NP membrane porosity, conversely, was higher than that of the reference membrane (68–75%).

### 3.3. Membrane Wettability Indicators

With respect to the liquid intrusion pressure, the pristine membrane (R) and all of the composite membranes have LEP_w_ values higher than 3 bars, excepting M12 and M25 with 12 wt% and 25 wt% MoS_2_, respectively, which have LEP_w_ values in the range of 2–3 bars. The risk of wetting is therefore higher for the M12 and M25 membranes compared with the other membranes; however, this parameter is not sufficiently informative to anticipate wetting because other parameters such as the operating conditions can also affect wetting.

The contact angle was lower for membranes with NPs than for the PVDF reference membrane, indicating variations in the surface hydrophobicity. Depending on the membrane, these variations can be slight or more significant, e.g., M2 and C2. However, there is no general trend with respect to the effect of the NP quantity on the hydrophobicity of the membrane surface.

### 3.4. Knudsen Permeability

**Figure 4:**
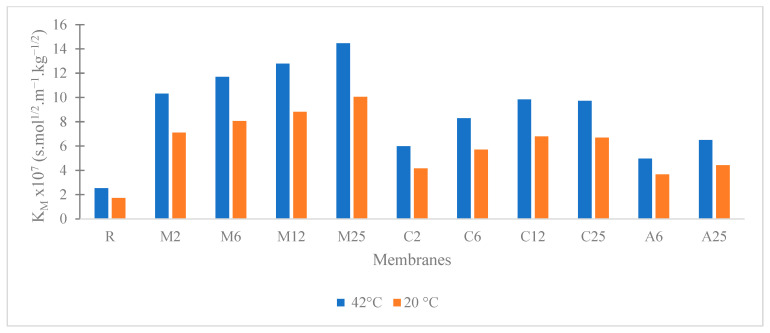
Knudsen permeability (K_M_) measurement of the prepared membranes according to the pressure variation method with pure water at 42 °C and corrected to 20 °C.

The Knudsen permeability K_M_ with pure water was determined at 42.5 °C and 20 °C for the reference membrane (R) and for each PVDF-NP membrane under normal MD conditions without exposure to sunlight. No leakage was observed for any of the prepared membranes. The results are presented in Figure 4.

The K_M_ value of pristine PVDF (R) is 1.7 × 10^−7^ s mol^1/2^ m^−1^ kg^−1/2^ at 20 °C. All composite membranes had a higher permeability than the reference membrane. In addition, the membranes with MoS_2_ were more permeable than the carbon black-based membranes. This is likely due to the membrane structure, as previously mentioned in the context of the morphology characterization. This result indicates that the incorporation of NPs positively affects the membrane permeability. The Knudsen permeability of tested membranes can be classified from low to high in the following manner: reference membrane (R) < membranes with Ag NPs (A6 and A25) < membranes with carbon black (C2–C25) < membranes with MoS_2_ (M2–M25).

In addition, for each photothermal material, K_M_ increases with increasing NP proportion in the PVDF membranes. K_M_ at 20 °C is multiplied by a factor 2.6 for Ag NPs and by factors of 4 and 6 for carbon black and MoS_2_, respectively, when the load varies from 0 (corresponding to the reference membrane) to 25%.

### 3.5. Evaporation Test

**Figure 5:**
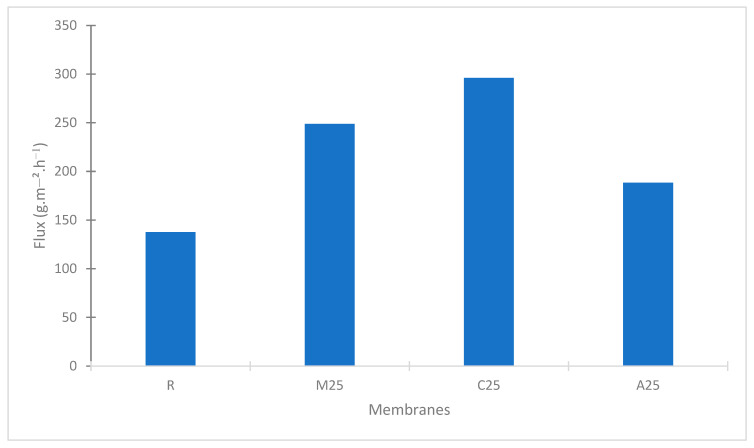
Water evaporation fluxes of the prepared membranes during a 24-h period.

The mass loss caused by evaporation was recorded for 24 h at room temperature (~21 °C). The evaporation fluxes at room temperature of the pristine and composite membranes are presented in Figure 5.

Compared with the flux of R (pristine PVDF), the flux of M25 (PVDF-25% MoS_2_) is 80% higher, the flux of C25 (PVDF-25% carbon) is 110% higher, and the flux of A25 (PVDF-25% Ag) is 36% higher. This verifies the photothermal effect of the NPs used in the membranes.

### 3.6. Permeate Fluxes Obtained during VMD Operation with a Saline Solution

**Figure 6:**
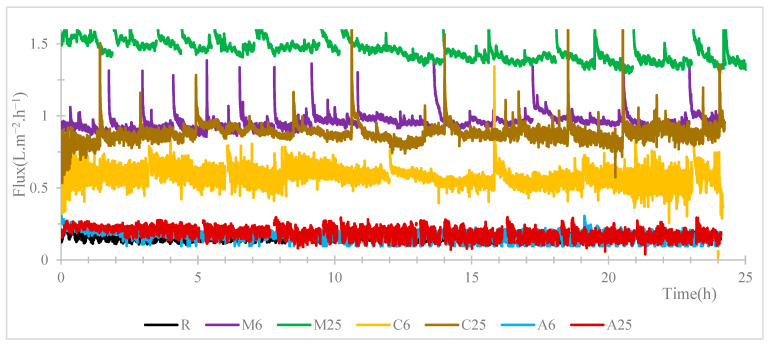
Permeate fluxes obtained during VMD operation with a saline solution during a 24-h period.

The permeate fluxes obtained during VMD operation with a saline solution as a feed for membranes R, and M, C and A for 6% and 25% NPs are represented on Figure 6. The lowest fluxes are achieved with the reference membrane and for the two membranes based on Ag-NPs. The highest fluxes are obtained for the two membranes based on Mos_2._ and the carbon black membranes present fluxes higher in between the one of MoS_2_ and Ag membranes. For both the carbon black and MoS_2_, the NP load increases the flux.

### 3.7. Wetting Characterization According to the DDTI Method

A complete set of data was obtained for the nine sampling zones in each type of membrane, and the results for the wetting indicator on each sample (at the local scale) are given in Table 6. Note that the results in area O_3_ for the M2 membrane could not be used because of a technical problem during sampling that led to possible salt contamination.

At the local scale (ω_p_ local), several observations can be made depending on the membrane.

For the pristine PVDF membrane without nanoparticles, only no wetting or surface wetting (in yellow) were observed regardless of the sampling zone. This means that the intrusion of liquid inside the pores was very limited. Consequently, this membrane was not significantly affected by wetting and the liquid did not intrude very far into the pores under the standard operating conditions used.

For membranes based on MoS_2_ (M2–M25) and on carbon black (C2–C25), the frequency of the no-wetting mechanism was greater than the frequency of the surface-wetting mechanism compared with the pristine PVDF membrane. Overall, the composite membranes were even less wetted than the PVDF membrane and displayed more homogeneous behavior.

For Ag membranes with a similar ratio of NPs, the four wetting mechanisms, even total wetting, can be observed in the different zones of the membranes. It appears that Ag-Np composite membranes are better able to wet deeply in some areas than composite membranes based on the other NPs (MoS_2_ and carbon black) and the reference membrane.

The global pore wetting ratio was obtained by averaging the pore wetting ratios obtained in the nine zones of each membrane, or the eight zones for which results were obtained in the case of the M2 membrane. The results are summarized in Table 7.

Overall, based on the values of the global pore wetting ratio (ω_p_ global) and the corresponding global wetting mechanisms at the scale of the membrane, only two mechanisms were observed for the R, M2–M25, and C2–C25 membranes during the 24-h testing period: no wetting and surface wetting. Surface wetting was observed for the reference membrane, whereas no wetting was observed for most MoS_2_-based and carbon black-based membranes. However, a more severe wetting behavior was observed for the Ag-based membranes regardless of the Ag load.

Overall, the membranes prepared with MoS_2_ and carbon black NPs are sustainable for PMD application and are not prone to wetting (at least in the short term). At the same time, they have a higher permeability compared with the reference PVDF membrane. However, PVDF membranes prepared with Ag NPs present partial wetting after 24 h of operation and are not a sustainable option for PMD under the operating conditions chosen here for membrane preparation and testing.

## 4. Conclusions

PMD is an emerging technology for desalination directly using solar energy; however, it requires specialized membranes that include photothermal NPs and can combine photothermal properties, permeability to water vapor, and wetting resistance. The application of this process for desalination at an industrial scale is feasible if cheap membranes can be developed.

Based on our previous work and experience concerning wetting detection methods for porous membranes and membrane preparation with included NPs in polymeric matrixes, four families of PVDF membranes based on different photothermal membranes were developed and compared (i.e., a polymeric membrane; membranes based on a promising semi-conductor, MoS_2_; carbon black; and metallic Ag) with different NP loads.

A full characterization of the membranes was performed, including an examination of the structural properties, their performances expressed in terms of the Knudsen permeability, and the photonic effect estimated according to the evaporation rate under fixed lightning, as well as the wettability indicators and the effective wetting characterization after MD operation with exposure to saline solutions.

The results summarized in Table 8 clearly show the advantages of MoS_2_ and carbon black-based photothermal membranes in comparison with PVDF membranes in terms of both the permeability increase and the less severe wetting mechanism, with lower wetting indicators in the short term. The results also indicate that these materials present multiple advantages in comparison with Ag. They are both much cheaper than this noble and rare metallic material and present higher permeabilities and less severe wetting mechanisms.

As a resource, the availability and lower cost of carbon black and its main advantages in comparison with the PVDF membrane likely render it a highly suitable option. However, further studies are required to decide between MoS_2_ and carbon black for sustainable PMD operation in desalination, notably studies on the optimization of the NP load, the membrane structure with only a surface photothermal deposit based on longer term testing of the wetting, and the fouling experienced when using the various membranes.

## Figures and Tables

**Table 1:** Photothermal materials and relative light/heat conversion mechanisms.

Material Family	Examples of Effective Materials [4,6,8]	Solar to Heat Energy Conversion Mechanism [3,7]
Metallic nanostructures	Gold, silver, aluminum, copper, and palladium in the form of nanoparticles or composites	Localized plasmonic heating
Inorganic semiconductors	TiO_x_, TiN, CuS, and MoO_x_	Electron-hole generation and relaxation
Carbon-based light-absorbing materials	Carbon nanotubes, graphene, graphene oxide (GO), reduced graphene oxide (rGO), carbon black (CB), and carbonized natural products	Thermal molecular vibration
Polymeric materials	Polypyrene (PPy)	Thermal molecular vibration

**Table 2:** Collodion composition corresponding to the target proportion of nanoparticles (NPs) in the membranes, (where X indicates M for MoS_2_ NPs, C for carbon black NPs, or A for Ag NPs.).

		For 100 g of Collodion			
Notation	Membrane Code	PVDF (g)	DMF (g)	NPs (g)	% NPs in Membrane
R	PVDF-REF	15	85	0	0%
X2	PVDF-2% NPs	15	84.7	0.3	2%
X6	PVDF-6% NPs	15	84	1	6%
X12	PVDF-12% NPs	15	83	2	12%
X25	PVDF-25% NPs	15	80	5	25%

**Table 3:** Pore wetting ratio (ω_p_) ranges and corresponding wetting mechanisms [22].

Pore Wetting Ratio (ω_p_)	Wetting Mechanism and Visualization of the Corresponding Color Codes
ω_p_ ≤ 1%	No wetting
1.1% < ω_p_ ≤ 10%	Surface wetting
10.1% < ω_p_ ≤ 90%	Partial wetting
ω_p_ > 90%	Total wetting

Green color = no wetting, yellow color = sufrace wetting, orage color = partiel wetting, red color = total wetting.

**Table 4:** Scanning electron microscopy (SEM) images of all composite membranes.

Membrane	SEM Image (Magnification1000×, HV 20 kV, WD: 25 mm, Scale 30 µm)
R PVDF-REF	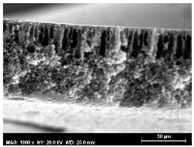
M2 PVDF-2% MoS_2_	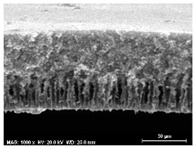
M6 PVDF-6% MoS_2_	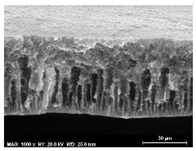
M12 PVDF-12% MoS_2_	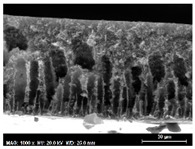
M25 PVDF-25% MoS_2_	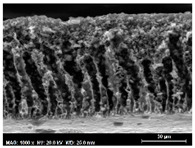
C2 PVDF-2% Carbon black	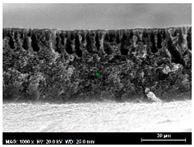
C6 PVDF-6% Carbon black	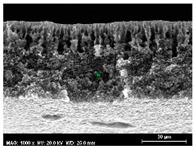
C12 PVDF-12% Carbon black	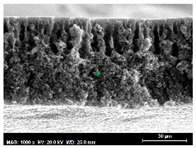
C25 PVDF-25% Carbon black	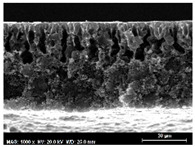
A6 PVDF-6% Ag	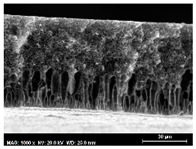
A25 PVDF-25% Ag	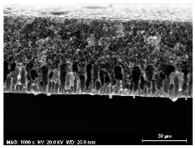

**Table 5:** Liquid entry pressure of pure water (LEP_w_), contact angle, thickness, and porosity characterization of all membranes.

Membrane	LEP_w_ (bars)	Contact Angle (°)	Thickness (µm)	Porosity (%)
R	>4	88	58	62
M2	>4	61	49	61
M6	3.6	85	50	60
M12	2.6	79	60	66
M25	2.8	80	61	62
C2	>4	60	49	62
C6	>4	80	48	57
C12	>4	85	53	62
C25	3	88	59	60
A6	>4	78	62	68
A25	4	72	60	75

**Table 6:** Pore wetting ratio at the local scale (ω_p_ local) for all membranes.

Membrane	ω_p_ local (%)								
	I_1_	I_2_	I_3_	M_1_	M_2_	M_3_	O_1_	O_2_	O_3_
R	0	0	3	0	0	8.3	0.6	0.9	1.9
M2	0	0	0	0	3	0	0	5	-
M6	0	8	0	0	0	0	0	0	0
M12	3	2	0	0	1	4	0	0	2
M25	0	0	0	4	0	0	0	1	4
C2	0	0	11	0	0	0	0	0	0
C6	0	0	0	0	0	0	0	0	0
C12	0	0	0	3	3	0	2	0	0
C25	1	0	0	0	0	0	0	0	3
A6	0	44	4	16	93	1	2	0	**7**
A25	0	82	95	0	0	0	0	0	**3**

**Table 7:** Pore wetting ratio at the global scale (ω_p_ global) for all membranes.

Membrane	ω_p_ Global (%)
R	1.6
M2	1.0
M6	0.9
M12	1.3
M25	1.0
C2	1.2
C6	0
C12	0.9
C25	0.4
A6	19
A25	20

**Table 8:** Summary and comparative view of the results obtained in this study.

Membrane	PVDF	PVDF-MoS_2_	PVDF-Carbon Black	PVDF-Ag
K_M_	Reference	+++ Increases with NP load	++ Increases with NP load	+ Increases with NP load
Evaporation rate	Reference	++	+++	+
Permeate flux during VMD with a saline solution	Reference	+++	++	=
Wettability	Reference	Increases with NP load	Slight increase with NP load	=
Wetting	Reference	Less wetting	Less wetting	Much more wetting
Cost	Low	Moderate	Low	Very high

## Data Availability

The data supporting reported results are available from the corresponding author upon reasonable request.
